# Usage of a Generic Web-Based Self-Management Intervention for Breast Cancer Survivors: Substudy Analysis of the BREATH Trial

**DOI:** 10.2196/jmir.2566

**Published:** 2013-08-19

**Authors:** Sanne W van den Berg, Esmee J Peters, J Frank Kraaijeveld, Marieke FM Gielissen, Judith B Prins

**Affiliations:** ^1^Department of Medical PsychologyRadboud University Nijmegen Medical CentreNijmegenNetherlands; ^2^Department of Medical OncologyRadboud University Nijmegen Medical CentreNijmegenNetherlands; ^3^IPPZICT & Consultancy for HealthcareUtrechtNetherlands

**Keywords:** usage evaluation, usage statistics, intervention adherence, user groups, exposure, Internet, Web-based intervention, breast cancer, log file analysis, website use

## Abstract

**Background:**

Generic fully automated Web-based self-management interventions are upcoming, for example, for the growing number of breast cancer survivors. It is hypothesized that the use of these interventions is more individualized and that users apply a large amount of self-tailoring. However, technical usage evaluations of these types of interventions are scarce and practical guidelines are lacking.

**Objective:**

To gain insight into meaningful usage parameters to evaluate the use of generic fully automated Web-based interventions by assessing how breast cancer survivors use a generic self-management website. Final aim is to propose practical recommendations for researchers and information and communication technology (ICT) professionals who aim to design and evaluate the use of similar Web-based interventions.

**Methods:**

The BREAst cancer ehealTH (BREATH) intervention is a generic unguided fully automated website with stepwise weekly access and a fixed 4-month structure containing 104 intervention ingredients (ie, texts, tasks, tests, videos). By monitoring https-server requests, technical usage statistics were recorded for the intervention group of the randomized controlled trial. Observed usage was analyzed by measures of frequency, duration, and activity. Intervention adherence was defined as continuous usage, or the proportion of participants who started using the intervention and continued to log in during all four phases. By comparing observed to minimal intended usage (frequency and activity), different user groups were defined.

**Results:**

Usage statistics for 4 months were collected from 70 breast cancer survivors (mean age 50.9 years). Frequency of logins/person ranged from 0 to 45, total duration/person from 0 to 2324 minutes (38.7 hours), and activity from opening none to all intervention ingredients. 31 participants continued logging in to all four phases resulting in an intervention adherence rate of 44.3% (95% CI 33.2-55.9). Nine nonusers (13%), 30 low users (43%), and 31 high users (44%) were defined. Low and high users differed significantly on frequency (*P*<.001), total duration (*P*<.001), session duration (*P*=.009), and activity (*P*<.001). High users logged in an average of 21 times, had a mean session duration of 33 minutes, and opened on average 91% of all ingredients. Signing the self-help contract (*P*<.001), reporting usefulness of ingredients (*P*=.003), overall satisfaction (*P*=.028), and user friendliness evaluation (*P*=.003) were higher in high users. User groups did not differ on age, education, and baseline distress.

**Conclusions:**

By reporting the usage of a self-management website for breast cancer survivors, the present study gained first insight into the design of usage evaluations of generic fully automated Web-based interventions. It is recommended to (1) incorporate usage statistics that reflect the amount of self-tailoring applied by users, (2) combine technical usage statistics with self-reported usefulness, and (3) use qualitative measures. Also, (4) a pilot usage evaluation should be a fixed step in the development process of novel Web-based interventions, and (5) it is essential for researchers to gain insight into the rationale of recorded and nonrecorded usage statistics.

**Trial Registration:**

Netherlands Trial Register (NTR): 2935; http://www.trialregister.nl/trialreg/admin/rctview.asp?TC=2935 (Archived by WebCite at http://www.webcitation.org/6IkX1ADEV).

## Introduction

### Background

A growing number of women survive breast cancer treatment [1]. The information need is high in these breast cancer survivors [2], and 40-49% of women turn to the Internet for information or support [3-6]. Most breast cancer survivors (70-80%) do not experience severely elevated levels of distress and are not in need of intensive therapist-led psychological treatment [7,8]. Therefore, self-guided Web-based therapeutic interventions [9] seem appropriate to provide easily accessible support to this large number of women at low health care costs. These unguided generic Web-based self-management interventions for breast cancer survivors are emerging and promising [10-12]. However, research data on the use of these type of Web-based interventions are scarce and inconclusive.

Better understanding of website use is an essential step in explaining how Web-based interventions produce behavior change and symptom improvement [13]. The technical usage statistics derived from a website are a representation of the individual processes by which participants use the intervention [14]. These statistics enable us to determine the real-life or observed usage and can be used to calculate adherence rates of Web-based interventions [15]. In addition, the evaluation of usage statistics (usage evaluations or logfile analysis) can reveal important design implications for more effective Web-based interventions [14].

Usage evaluations have been a relatively new area of interest in Internet intervention research. The newly proposed Consolidated Standards of Reporting Trials on eHealth applications (CONSORT-EHEALTH [16]) include the recommendation to report usage parameters. However, practical guidelines are scarce with regard to which usage parameters are preferred to measure observed usage [14,17]. Systematic reviews on the use of Web-based interventions reported a variety of usage statistics, which could be classified into (1) frequency of use (ie, frequency of logins or visits, mean logins during intervention, days on which intervention was visited), (2) duration (ie, length of time logged in), and (3) activity (ie, page views or number of unique pages visited, chapters, or modules completed) [15,18,19]. This multiplicity of usage statistics was also found in usage evaluations of Web-based interventions specifically designed for cancer survivors [10-12]. Deduced from these research findings, at least *frequency, duration,* and *activity* should be measured as usage statistics for evaluating the observed usage in Web-based interventions [19].

Evaluating the observed usage is especially important in generic fully automated Web-based interventions. The generic content of these interventions is offered to a heterogeneous group of users, and no professionals are available to tailor the intervention to meet the needs of each individual user. Therefore, we propose the term “self-tailoring” to refer to the degree the user tailors the intervention and selects the content that suits his/her personal situation or needs.

In addition to reporting the observed usage, it is of equal importance to report the intended usage [16,20]. The intended usage is defined prior to evaluation of the observed usage and refers to “the extent to which the developers of the intervention felt that the intervention should be used to achieve the desired effect” [20]. Evaluation of both observed and intended usage can provide insight into whether the intervention was used as envisioned. By comparing the intended usage to the observed usage, a priori defined types of users or user groups can be examined.

### Objective

Summarizing, the use of novel generic Web-based interventions is largely unknown and practical guidelines for technical usage evaluations are lacking. Usage evaluations are especially of added value with regard to unguided generic fully automated interventions. It is hypothesized that the use of these generic interventions is more individualized and that users apply a large amount of self-tailoring to the intervention content. Therefore, the present study aims to (1) gain insight into which usage parameters are needed to meaningfully evaluate the usage of generic fully-automated Web-based interventions, by (2) investigating in what amount and how breast cancer survivors use a generic Web-based self-management intervention. Our final aim is to (3) propose practical recommendations for researchers and information and communication technology (ICT) professionals who aim to design and evaluate the use of similar Web-based interventions.

## Methods

### Participants

This study focused on the analyses of all participants randomly allocated to the intervention group of the BREAst cancer ehealTH (BREATH) randomized controlled trial (RCT). This two-arm RCT evaluated the efficacy of a Web-based self-management intervention for breast cancer survivors compared to care as usual. Full details of the trial design, eligibility criteria, and patient recruitment have been described in the study protocol [21]. All participants were (1) women, (2) survivors of primary non-metastatic breast cancer, (3) between 2 and 4 months post treatment, (4) Dutch-speaking, with (5) direct access to a computer with Internet connection, and (6) in possession of an email address.

### Intervention

The unguided fully automated Web-based self-management intervention BREATH is based on cognitive behavior therapy (CBT) and aims to foster emotional adjustment after completion of primary curative breast cancer treatment. For a detailed description of the intervention and development process, we refer to the study protocol [21]. In this study, only the details necessary to comprehend the technical usage evaluation are reported. The BREATH intervention covers four phases of recovery after breast cancer, namely Looking Back, Emotional Processing, Strengthening, and Looking Ahead (for a screenshot see Multimedia Appendix 1). The intervention has a fixed structure with each phase covering 4 weeks. Intervention ingredients (104 in total) include Information (26 scripts), Assignment (48 tasks), Assessment (10 tests), and Video (20 ingredients with thematically clustered video clips extracted from recorded interviews). As a result of the generic character of the intervention, the usage is ad libitum: participants are free to select the intervention ingredients that they find useful or that apply to their personal situation. The first intervention ingredient of the intervention is a self-help contract to stimulate adherence.

The intervention is fully automated following a stepwise weekly access. Each week, on Monday, a reminder email is sent that new information is available. Participants can retrospectively access intervention ingredients of previous weeks but do not have access to forthcoming weeks. In addition to the intervention ingredients, Distress thermometers [22] are available to track the course of experienced distress over the 4-month intervention. Distress thermometers are optional and can be completed with a maximum of 1 per day. Email was used only for reporting technical problems with the website. Based on the session duration of face-to-face CBT, the intended session duration was a maximum of 1 hour per week. The BREATH intervention was developed by a clinical psychologist (JBP) and an eHealth researcher (SWvdB) in close cooperation with ICT professionals (JFK). A multidisciplinary reading committee (including patients, oncology professionals, cancer patient organizations, and patient advocates) reviewed and provided feedback on the thematic content of the intervention [21].

### Usage Data Retrieval

The BREATH intervention was developed within the eHealth application myTherapy (IPPZ), designed for online information, communication, and treatment in health care. User-initiated activity in the intervention was determined by monitoring https-server requests. Such requests could be database reads or writes and were logged for various purposes. Database reads could be logged for, for example, logins or opening an intervention ingredient, and database writes for, for example, adding text to an assignment. In most cases, database reads and writes included a timestamp derived from time of the server request. In some cases, timestamps could be combined to calculate duration. Data were retrieved using logs and database tables used by the Web application myTherapy. Information regarding specific activity (eg, intervention ingredients and internal mail) and user profiles (eg, avatars) was saved or logged to display information to users of myTherapy. For each individual user, user-initiated activity was monitored for a period of 16 weeks.

The occasional absence of data (ie, seconds in log out, precise click path) was in most cases due to design decisions focused on an operational Web application rather than on research purposes. In only a few cases, logging of usage data (eg, logins, login duration) was inaccurate due to rare combinations of, for example, archaic browser type, browser privacy settings, and company network settings. To overcome the problem of patients who forgot to log out, patients were automatically logged out after 30 minutes of inactivity on the website (measured as a 30-minute absence of server requests). During the study period, myTherapy was updated, varying from minor updates (bug fixes) to major updates (minor template changes, improving planning and user interface). In particular, between September 2011 and March 2012, myTherapy suffered irregular short periods of downtime. The total downtime added to less than 1% of the total time.

### Outcome Measures: Usage

#### Frequency, Duration, and Activity

The amount of use of the BREATH intervention was measured with the usage statistics of frequency, duration, and activity. *Frequency* was operationalized as the number of logins per patient during the 4-month period of the intervention. A login was defined as every time a patient signed in to the website for a minimum of 1 minute because no seconds were recorded concerning the logout time. Two types of *duration* were analyzed: session duration and total duration. Session duration was defined as the time (start-stop) of one login in minutes: the time between logging in and logging out. Total duration was the sum of all sessions per patient in minutes. *Activity* was defined as the number of opened intervention ingredients (ie, scripts, tasks, tests, videos) per patient, with a maximum of 104.

To gain insight into how patients used the intervention, we also calculated the distribution (ie, videos, assignments, information, assessments) of the total opened intervention ingredients per patient, using an avatar (yes/no), the number of Distress Thermometers completed, and the number of emails sent to report technical problems with the website, and whether they opened the self-help contract at the beginning of the intervention (yes/no) and signed the contract by filling in their name and the date (considered as actively using the self-help contract). Following the use of 45 assignments and assessments, users were asked whether they perceived these ingredients as useful. This self-reported usefulness was optional to fill in at the end of the assignment or assessment and scored as useful (1), not useful (2), or not filled in (0). For each participant, the proportion of opened ingredients perceived as useful, not useful, or not filled in was calculated.

Last, to evaluate whether the fixed structure was used as such, we calculated how many intervention ingredients were opened in the phase they were originally planned. To analyze on which days participants log in to the intervention during the week, each login was coded into a nominal variable representing Sunday-Saturday (1-7). Results on how the intervention was used were reported for low and high users.

#### Intervention Adherence and Nonusage Attrition

In this study, intervention adherence solely referred to the extent to which participants were exposed to the content of the intervention, not adherence to research protocol assessments (eg, filling out questionnaires) [20,23,24]. In addition, nonusage attrition [24] (or nonadherence) referred to the proportion of participants who stopped using the intervention over time. In Internet intervention research, there is a lack of agreement about which definitions and usage statistics should best be used to measure adherence or nonusage attrition [15]. In the current study, intervention adherence was defined as user persistence or *continuous usage*: the proportion of patients who started using the intervention and continued to log in (at least once) during all four phases. Nonusage attrition was defined as *intermittent usage*: the proportion of patients who did not log in during all four phases of the intervention. Continuous and intermittent usage were measured based on frequency of logins. For each participant, it was calculated in which phases (1-4) and weeks (1-16) logins took place.

#### User Groups

To evaluate how participants used the intervention differently, user groups were calculated by comparing the intended usage to the observed usage. The minimal intended frequency of logins as formulated by the developers of the BREATH intervention was a minimum of 10 times over the course of the intervention and was based on the frequency of face-to-face CBT. Also, the intervention ingredients of 1 week should take a maximum of 1 hour to complete. The minimal intended activity was opening a minimum of 50% of the total 104 intervention ingredients, because not all ingredients of the generic intervention will apply to the personal situation of every user. Table 1 gives an overview of the classification of four user groups based on minimal intended frequency and activity. To calculate user groups, the observed frequency and activity was cross-tabulated within a 4x4 matrix of intended frequency and activity.

### Outcome Measures: Other

#### Baseline Survey

At baseline, before randomization, participants of the BREATH RCT filled in an online survey with questions concerning sociodemographic characteristics (eg, age, marital status, children, education, employment status), medical characteristics (eg, type of adjuvant therapy, use of hormonal therapy), and psychological questionnaires (for a full overview see [21]). Education was measured using a 7-point scale [25] ranging from primary education not finished (1) to master’s degree (7). For this study, with regard to psychological questionnaires, only the Hospital Anxiety and Depression Scales (HADS) [26] was reported to assess baseline general distress [27]. The total score of the HADS (HADS-T) has demonstrated good reliability and validity in oncology patients [28,29]. A HADS-T of ≥11 represented elevated levels of distress indicative for mental disorders [30].

#### Evaluation Survey

After the intervention (4 months after baseline), participants completed an online survey including an evaluation of the intervention. For the intervention evaluation, two single-item measures were examined: overall satisfaction (“Which grade would you give to the overall intervention?”) and user friendliness (“Which grade would you give to the user friendliness of the intervention?”). These measures were scored on a 10-point scale ranging from 1 (very bad) to 10 (very good). For qualitative results, participants were asked to report points for improvements of the intervention.

#### Statistical Analyses

All analyses were performed using SPSS 20. For all nondescriptive outcome measures, the amount, the percentage, and the Wilson confidence interval (CI) were reported. Usage statistics, sociodemographic, and medical characteristics were not normally distributed as indicated with the Kolmogorov-Smirnov test (<0.05) and therefore were first analyzed using nonparametric tests. To facilitate interpretation, parametric tests were reported, since results did not differ from nonparametric tests. Pearson’s correlation coefficients were reported between technical usage statistics (ie, frequency, session duration, total duration, and activity) and between usage statistics and the patient characteristics. To assess differences between user groups, *t* tests, Pearson’s chi-square tests, and Fisher exact tests were conducted. A two-sided alpha=.05 level of significance was used for all analyses.

**Table 1:** Classification of user groups based on minimal intended frequency and activity.

	Minimal intended frequency	Minimal intended activity
Nonusers	0	0
Low users	1	1%
Intended users	10	50%
High users	17	75%

## Results

### Sociodemographic and Medical Characteristics

Seventy participants were included in the study sample and had been in the position to log in to the BREATH intervention for a period of 4 months. Usage statistics were recorded from November 2010 until August 2012. Of all participants, mean age was 50.9 (SD 8.31), the mean education level on a 7-point scale was 5 (SD 1.63), and 1 participant did not have Dutch nationality. Forty percent of the patients were employed (28/70), 37% (33/70) received full or partial disablement insurance or were on sick leave, 83% of the participants (58/70) were married or living together with a partner, and 87% of the participants (61/70) had children. All participants were treated with surgery and adjuvant therapy for breast cancer: 27% (19/70) received only chemotherapy, 4% (3/70) received only radiotherapy, and 69% (48/70) received both chemotherapy and radiotherapy. In addition, 66% of the participants (46/70) received hormonal therapy during the intervention period. At baseline, 27% of the participants (19/70) experienced elevated levels of distress based on HADS-T≥11.

### Frequency, Duration, and Activity

Participants demonstrated a large variability in intervention usage over the 4 months in which the intervention was available. Frequency ranged from 0 to 45 logins (mean 11, SD 7), and 10% (7/70) of the participants never logged in to the intervention. Total duration per participant ranged from 0 to 2324 minutes (38.7 hours), with a mean total duration per participant of 337.2 minutes (SD 163.7), which equals 5.6 hours. The mean of the average session duration per patient was 24.7 minutes (SD 16.1). *Activity* ranged from opening none to all intervention ingredients, with a mean of opened intervention ingredients per participant of 49.9 (SD 42.8), and 13% (9/70) of the participants never opened an intervention ingredient. Frequency was positively correlated with total duration (*r*=.83), session duration (*r*=.40), and activity (*r*=.84), and high activity was associated with a longer total (*r*=.75) and session duration (*r*=.55). All correlations were significant on the *P*<.001 level. Correlations between total and session duration were not calculated because total duration was calculated with session duration.

With regard to how patients used the intervention, 69% of the participants (48/70; 95% CI 56.97-78.24) opened the self-help contract, and 17% (12/70; 95% CI 10.09-27.62) made use of an avatar. Of all participants, 63% (44/70) filled in at least one Distress Thermometers: median 2 and a maximum of 13. Seven participants sent emails to the researcher concerning technical problems with the intervention. There were significant differences between the login days (*P*<.001), with 28% (CI 24.80-31.17) of all logins (n=757) being on the day the weekly reminder was sent (Monday).

### Intervention Adherence and Nonusage Attrition

**Figure 1:**
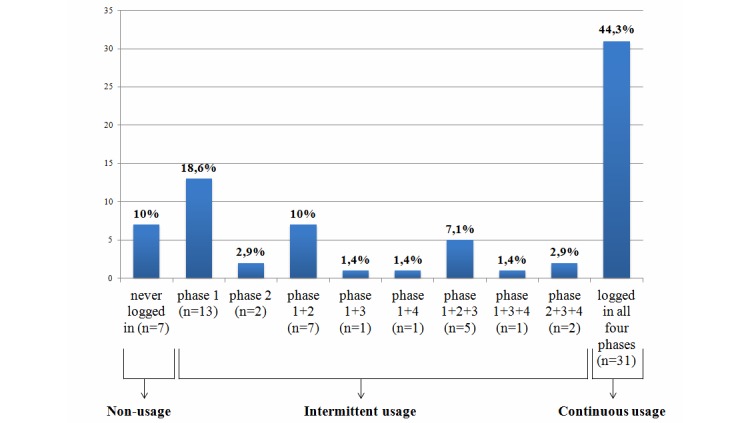
Continuous usage and intermittent usage based on logins during 4 intervention phases (n=70).

Figure 1 shows the intervention adherence (defined as continuous usage) and nonusage attrition (defined as intermittent usage) based on logins during the four intervention phases. Of the total sample, 31 participants logged in to the intervention website during all four phases, resulting in a continuous usage of 44.3% (95% CI 33.2-55.9). Of these participants, only 6 logged in during all 16 weeks of the intervention.

Seven participants (10%) never logged in to the website and were thus never exposed to the intervention content. Intermittent usage was 45.7% (32/70): 13 participants (18.6%) only logged in during the first phase, and 2 participants (2.9%) only logged in during the second phase. Nine users (12.6%) logged in during two of the four phases, and 8 users (11.4%) logged in only during three phases.

### User Groups

This study showed three user groups based on the comparison of intended versus observed frequency and activity: 9 nonusers, 30 low users, and 31 high users. Seven nonusers never logged in, and 2 nonusers logged in once but did not open any intervention ingredients. Only 2 participants met the classification of intended user as specified beforehand. Not being considered a substantial group, these 2 intended users were listed as high users. Low and high users differed significantly on frequency (*P*<.001), total duration (*P*<.001), session duration (*P*=.009), and activity (*P*<.001). Low users logged in with an average of 3.6 times (SD 2.6) over the course of the 4-month intervention and had a mean session duration of 23.5 minutes (SD 12.3). The mean total duration that low users spent on the website was 81.1 minutes (SD 75.5) in which they opened a mean of 18.8/104 ingredients (SD 17.2). High users logged in with an average of 21 times (SD 9), which is more than once a week, and had a mean session duration of 32.8 minutes (SD 14.4). The mean total duration that high users spent on the website was 682.7 minutes (SD 443 minutes), which equals 11 hours and 22 minutes. During this time, high users opened on average 91% of all intervention ingredients (mean 94.5/104 ingredients, SD 12.8).

Group characteristics of the three user groups are reported in Table 2. On baseline distress, sociodemographic, and medical characteristics, no significant differences were found between nonusers versus users (low and high users), and low users versus high users.

With regard to how the intervention was used, high users completed significantly more Distress Thermometers (mean 5, SD 2.5) compared to low users (mean 1, SD 1.5; *P*<.001). In addition, all high users (100%; 31/31) opened the self-help contract at the beginning of the intervention, versus 57% (17/30) of the low users (*P*<.001). Following the opening of the self-help contract, 84% (26/31) of the high users also signed the contract versus 53% (9/17) of the low users (*P*<.001).

Self-reported usefulness was gathered for the majority of the intervention ingredients that required active input from users (assignments and assessments). The proportion of opened ingredients perceived as useful was higher in high users (mean 67%, SD 21%) compared to low users (mean 44%, SD 25%; *P*<.001). High users filled in the self-reported usefulness significantly more often than low users (mean proportion not filled 16%, SD 17%, versus mean 36%, SD 29%; *P*=.003). The proportion of opened ingredients reported as not useful was low and did not differ between high users (mean 18%, SD 18%) and low users (mean 21%, SD 20%; *P*=.557).

**Figure 2:**
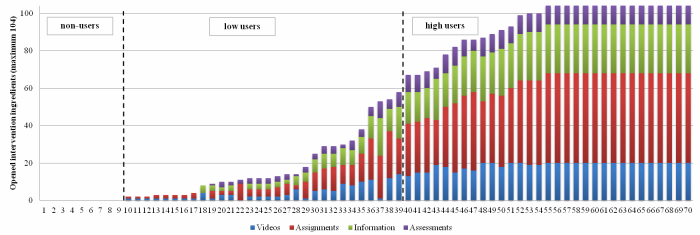
Distribution of total opened intervention ingredients per participants (n=70).

With regard to following the fixed structure, low users opened 19.7% of the intervention ingredients in a later phase than the ingredients were planned. High users followed the structure more and opened only 5.7% of the intervention ingredients in a later phase. The standard intervention distribution of the 104 ingredients was 46% assignments, 25% information, 19% videos, and 10% assignments. Figure 2 displays the distribution of intervention ingredients for each participant. Both low and high users did not show a strong preference in the type of opened intervention ingredients, for example, opening only videos. The proportion of opened assignments (40% vs 45%; *P*=.178), information (26% vs 25%; *P*=.850), and videos (21% vs 20%; *P*=.653) did not differ between low and high users. Low users opened proportionally more assessments compared to the high users (15% vs 10%; *P*=.036). However, this was related to the fact that all assessments were in the first two phases and low users opened predominantly ingredients in these first phases of the intervention. Last, high and low users did not differ on using an avatar or sending emails to the researcher about technical problems.

**Table 2:** Group characteristics (sociodemographic, social, medical) and baseline distress of the three user groups (n=70).

		Nonusers (n=9)	Users (n=61)		*P* value
Characteristics			Low users (n=30)	High users (n=31)	Low vs high users
Age, mean (SD)		51.9 (7)	51.83 (8.73)	49.7 (8.3)	.33^b^
Education (1-7), mean (SD)		5 (1.3)	4.9 (1.5)	5.2 (1.3)	.36^b^
Married/cohabiting, n (%)		6 (66.7)	27 (90)	25 (80.6)	.47^c^
Children, n (%)		8 (88.9)	28 (93.3)	25 (80.6)	.26^c^
**Employment**					
	Paid job, n (%)	2 (22.2)	14 (46.7)	12 (38.7)	.53^d^
	Disablement insurance act or sick leave, n (%)	5 (55.6)	10 (33.3)	18 (58.1)	.053^d^
**Adjuvant treatment**					
	Both chemotherapy and radiotherapy, n (%)	6 (66.7)	22 (73.3)	20 (64.5)	.46^d^
	Hormonal therapy, n (%)	8 (88.9)	16 (53.3)	22 (71)	.16^d^
**Baseline distress**					
	HADS-T^a^baseline, mean (SD)	10.6 (11.1)	8.6 (5.8)	9.1 (6.1)	.75 ^b^
	HADS-T≥ 11, n (%)	4 (44.4)	9 (30)	10 (32.3)	.85^d^

^a^HADS-T=Hospital Anxiety and Depression Scale-total score.

^b^Independent samples *t* test.

^c^Fisher’s exact test.

^d^Pearson chi-Square.

### Evaluation Survey

Post-intervention evaluation surveys were filled in by 20 low users and 30 high users. Two nonusers erroneously filled in evaluation surveys, but since they were never exposed to the intervention, these were left out of the analyses. High users evaluated both overall satisfaction (mean 7, SD 1.20 vs mean 5.75, SD 2.20; *P*=.028) and user friendliness (mean 7.27, SD 1.34 vs mean 5.58, SD 1.18; *P*=.003) significantly higher than low users. Twenty-five participants (11 low users and 14 high users) actively stated points for improvements to the intervention. The top three points for improvements were (1) possibility to get access to the intervention sooner after completion of breast cancer treatment (6/25, 24%), (2) lack of practical information (eg, on prostheses, wigs, bras; 4/25, 16%), and (3) poor user friendliness of logging in (security code sent to mobile phone; 3/25, 12%).

## Discussion

### Summary

The current formative usage evaluation of a self-management website for breast cancer survivors illustrated the supposed diverse and individualized usage of generic fully automated Web-based interventions. Evaluation of only the amount of usage on group level did not provide a valuable representation of the real-life exposure to the generic self-management intervention. Usage data on how the intervention was used proved to be informative and revealed that 44.3% of the women continued using the BREATH-intervention over the 4-month period. Also, the comparison of intended versus observed usage showed three different user groups. A small proportion of participants were never, or only once, exposed to the intervention and were classified as nonusers. While the intended user group proved to be nonsubstantial, two equally large groups of active users were defined: low users and high users. Apart from the significant differences in usage statistics, low and high users were found to have a distinctive way of how they used the intervention. High users had a more homogeneous and consistent usage compared to low users. High users exceeded the intended frequency and activity, signed the self-help contract at the beginning of the intervention, and followed the fixed time-locked structure of the intervention. Although technical usage statistics did not provide information on the amount of self-tailoring users applied after they opened intervention content, data on self-reported usefulness showed that high users perceived the majority of opened intervention ingredients as useful. User groups did not differ in pre-intervention distress, sociodemographic, or medical characteristics.

The choice, or technical availability, of usage statistics plays a crucial part in usage evaluations and poses hazards to misinterpretations. For example in this study, solely based on the finding that high users opened almost all intervention ingredients could lead to the premature conclusion that all ingredients were useful to these participants. The fact that on group level, no preferences were found in opening intervention ingredients could add to this misinterpretation. However, based on technical usage data, it was not possible to conclude that high users valued all ingredients equally, since data on re-opening intervention ingredients were lacking. Data on self-reported usefulness provided this missing information and proved to be essential in making conclusions about how users self-tailored the content of this generic self-management intervention. In contrast to low users, high users consistently reported about the usefulness of intervention ingredients, and they perceived the majority of opened intervention content useful. Therefore, we concluded that high users actively used the full intervention content.

We also concluded that high users self-tailor logins to their own timetable instead of logging in during each intervention week. This was based on the finding that only 6 high users logged in during all 16 weeks. This sheds new light on the mean frequency of logins of 21 times of the high users. Apparently, high users do not log in during some weeks but catch up during the next week by returning to the website several times that week. Combined with the knowledge that high users follow the phase-structure when it comes to opening intervention ingredients in the planned phase, this might imply that time-locks can be broader in the future. Session duration was around 30 minutes in both low and high users and was lower than the maximum intended session duration of 1 hour, which implies that the natural session duration of the BREATH-intervention is half an hour.

### Intervention Adherence

As a result of the lack of agreement about how best to define and measure adherence, we have chosen to define intervention adherence as continuous usage based on frequency of logins. In order to be transparent, we consistently reported “continuous usage” throughout the current manuscript or provided our operationalization in addition to adherence: “intervention adherence (continuous usage)”. In a systematic review, Donkin et al [15] found that most studies on Web-based interventions reported adherence based on frequency of logins. However, it is recommended to use a composite measure encompassing a variety of usage statistics for the calculation of adherence [15]. High correlations found between frequency, total duration, and activity in the present study suggest that these three usage statistics measure a similar construct of continuous usage and are therefore interchangeable in analyses of adherence in this study. Whether they are also interchangeable in the analyses of effectiveness needs further research, since Donkin et al [15] found that activity (defined as completion of modules) was most consistently related to outcomes in psychological health interventions. Confirmed by other studies [31], in the current usage evaluation duration was found to be the least precise and therefore least reliable usage statistic. Since it is unknown what users do when a website is opened on their computer screen, time spent on a website provides the least reliable estimation of exposure to an intervention content.

Information on both adherence and nonusage attrition can be similarly informative in future evaluations of effect. Previous research has demonstrated that nonadherers can benefit equally as adherers from the intervention content they completed [32]. In the current study, it is possible that the participants who logged in continuously or intermittently during three out of four phases, experienced an early effect, which made further use of the intervention redundant. Different factors may predict intervention adherence in Web-based interventions [13], such as support provided by a therapist or coach [18,33], intervention characteristics, being studied in the context of a RCT design, a high frequent intended usage, and the use of persuasive technology [20]. Sending email reminders is part of persuasive technology [34]. The positive influence of sending weekly email reminders on intervention adherence (in the current study defined as continuous usage) was confirmed by the fact that 28% of all logins were on the same day the email reminder was sent. Email reminders were standard, but every month the reminder contained a preview of the intervention content of the upcoming 4 weeks, which might also have had a beneficial effect on revisiting the intervention [35].

### Predictors of Usage

In this study, user groups only differed in usage statistics, which is how they were classified. With regard to how the intervention was used, high users signed the self-help contract more often and reported more consistently on the usefulness of ingredients compared to low users. However, in the current study we lacked data to know the causality of these findings. At this moment, we do not know whether signing the self-help contract and reporting usefulness are predictors of high use, or whether high use predicts signing the self-help contract and reporting usefulness. More research is needed to determine whether and how intervention characteristics (such as a self-help contract) or user characteristics (such as motivation, positive expectations) can influence high usage.

In addition, no specific sociodemographic, medical or personal characteristics were found that distinguished between user groups, supporting our hypothesis that the present generic fully automated intervention could be acceptable for a broad range patients. However, this also led to a lot of unanswered questions about possible predictors of usage. It is possible that other characteristics not taken into consideration in the present study predict who is going to be a low or high user. For example, information on pre-intervention needs was lacking. Although distress was not related to the observed usage, distress screening does not uncover unmet needs in posttreatment cancer survivors [36]. Other possible predictors of usage could be computer experience, social support, or illness burden. In a Web-based illness management support system for breast and prostate cancer patients (WebChoice), the level of computer experience proved to be a predictor of use, whereas low social support and high illness burden were associated with high use of specific intervention components [37]. Another explanation for the absence of predictors could be that the usage behavior itself predicts whether users continue to use the intervention or do not log in again.

### Pitfalls and Limitations

The most important pitfall of the current study was the absence of usage data on re-visiting or re-opening intervention ingredients due to design decisions focused on the intervention website being operational. As a result, we lacked technical usage information on patient preferences of certain types of intervention ingredients after their first opening. Data on self-reported usefulness provided nontechnical data on this matter and allowed us to make some statements about self-tailoring.

The current study also lacked essential qualitative knowledge about reasons to stop or continue using the intervention. For example, in this study overall satisfaction and user friendliness evaluation of the intervention was higher in high users, but the causality of this finding needs further qualitative investigation. Stopping with the intervention might be negatively related to characteristics of the website (eg, user friendliness, appearance), the content of the intervention (eg, did not meet the patients needs), or the patient (eg, too burdensome, concurrent life events).

### Recommendations for Researchers and ICT Professionals

Based on the pitfalls encountered in the current study we formulated the following recommendations for researchers and ICT-professionals conducting usage evaluations of generic fully automated Web-based interventions. First, choose usage statistics that give insight into the amount of self-tailoring that participants apply to the intervention content and structure. This implies to record both singular usage statistics (frequency, duration, activity) and composite usage statistics (time spent per ingredient, click-patterns, re-opening, or span of use [14]). Second, combine technical usage statistics with self-reported usefulness to gain additional information on specific intervention components. The question of whether an intervention component is useful or not is easily implemented at the end of each component and takes little effort for participants. In case of missing technical data, self-reported usefulness can provide valuable insight in the amount of self-tailoring applied by users. Third, combine technical usage statistics with qualitative measures (such as semistructured telephone interviews or online focus groups) for a comprehensive usage evaluation. Fourth, conduct a pilot usage evaluation with a variety of usage statistics as a fixed step in the iterative development process of Internet interventions. This way, decisions can be made about which usage statistics should meaningfully be taken into account, or left out, in the final evaluation of usage. Last, gain insight into the rationale of recorded and nonrecorded usage statistics. Researchers with basic knowledge of ICT combined with ICT professionals with basic knowledge about conducting research facilitate effective communication and clear agreements about usage evaluations.

### Conclusion

This study underscores the added value of evaluating usage statistics of generic Web-based interventions as a realistic estimation of exposure to intervention content. To the best of our knowledge, the present study gained first insight into the design of technical usage evaluations of generic fully automated Web-based interventions. Overall, and in concordance with research on more interactive eHealth applications [38], results suggest that investigating how generic fully automated Web-based interventions are used is far more informative than the amount of exposure. Usage statistics should be chosen accordingly. Further, it is recommended to collect both singular and composite usage statistics, include self-reported usefulness, and to pilot test a variety of usage statistics to aid decision making of meaningful usage parameters. Last, shared knowledge about ICT and conducting research is helpful in developing a meaningful rationale of technically recorded usage statistics of generic Web-based interventions.

## Multimedia Appendix 1

Screenshot of the Web-based non-guided self-management intervention BREATH (in Dutch).

## Abbreviations

BREATH: BREAst cancer eHealth
CBT: cognitive behavior therapy
HADS: Hospital Anxiety and Depression Scale
HADS-T: Hospital Anxiety and Depression Scale total
ICT: information and communication technology
RCT: randomized controlled trial
