# Supplementary figures and images for: Usage of a Generic Web-Based Self-Management Intervention for Breast Cancer Survivors: Substudy Analysis of the BREATH Trial
Source: J Med Internet Res. 2013 Aug 19;15(8):e170. doi: 10.2196/jmir.2566 (PMC3758022; doi:10.2196/jmir.2566)

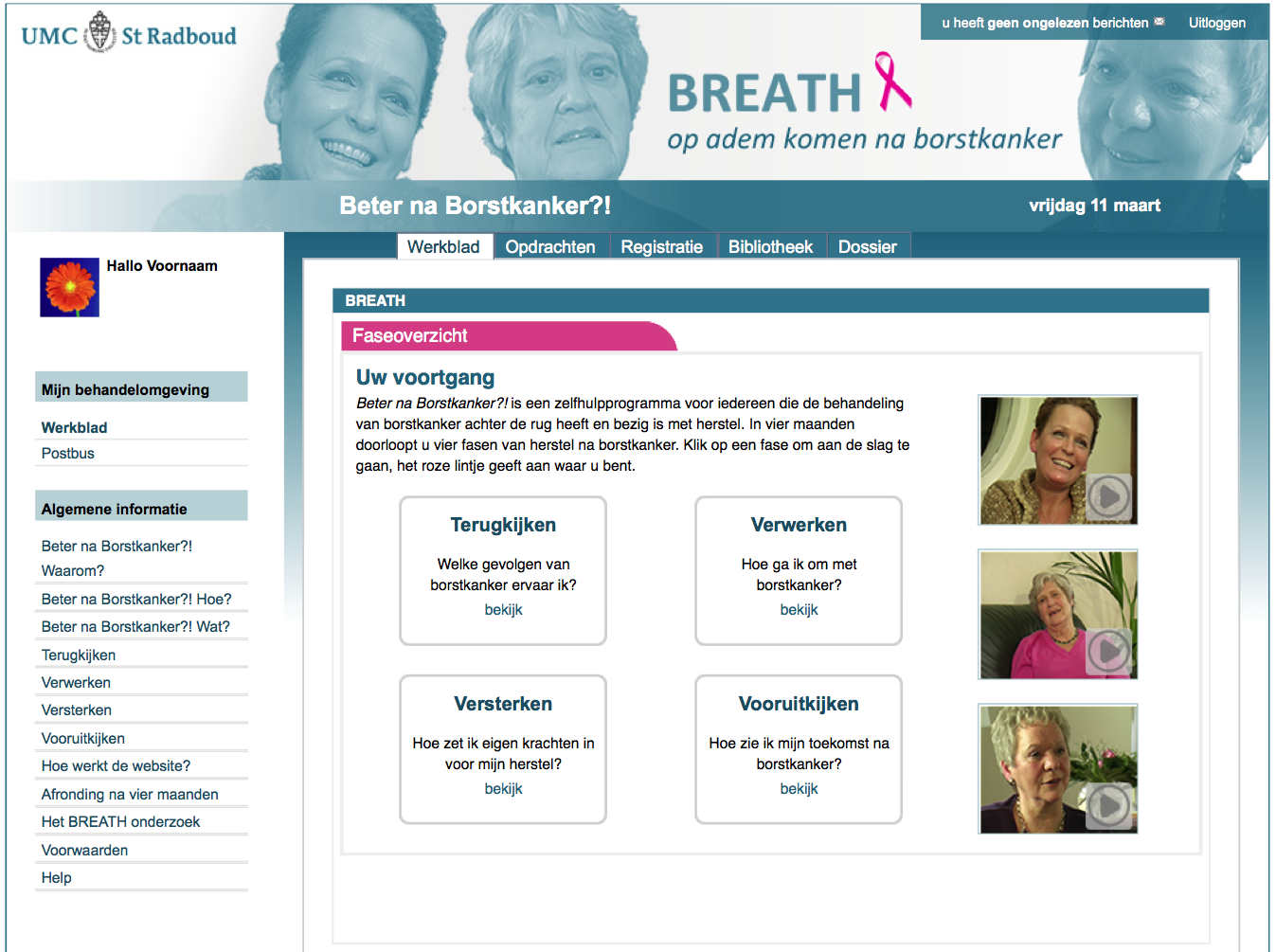

Supplement: Supplementary file 1 [file jmir_v15i8e170_app1.png]
